# Multimodal Imaging of Mediastinal Epithelioid Hemangioendothelioma: Two Case Reports

**DOI:** 10.2174/0115734056360811250516114535

**Published:** 2025-05-26

**Authors:** Tong Chen, Yapeng Sun, Mengsu Zeng, Mingliang Wang

**Affiliations:** 1 Department of Radiology, Zhongshan Hospital, Fudan University, Shanghai, China; 2 Department of Radiology, The Second Affiliated Hospital of Soochow University, Suzhou, China; 3 Department of Interventional and Vascular Surgery, Suzhou Research Center of Medical School, Suzhou Hospital, Affiliated Hospital of Medical School, Nanjing University, Suzhou, China; 4 Department of Radiology, Shanghai Geriatric Medical Center, Shanghai, China

**Keywords:** Epithelioid hemangioendothelioma, Mediastinal tumor, Superior vena cava, Multimodal imaging, Vascular neoplasm, Computed tomography

## Abstract

**Introduction:**

Epithelioid Hemangioendothelioma (EHE) is a rare vascular neoplasm that typically occurs in the bone, soft tissue, liver, and lung but rarely in the mediastinum. Multimodal imaging of EHE is poorly understood, often leading to misdiagnosis as other mediastinal tumors.

**Case Presentation:**

Two female cases with incidental mediastinal masses were retrospectively analysed, focusing on multimodal presentations. For both cases, CT studies showed well-defined, low-density oval masses in the right anterior superior mediastinum with the Superior Vena Cava (SVC) invasion. Intralesional punctate calcifications were observed in Case 2. MRI revealed hypointense masses on T1WI and slightly hyperintense on T2WI, with partial diffusion restriction on DWI. Case 1 had mild enhancement, while Case 2 had significant enhancement. PET-CT showed significant FDG uptake with maximum standardized uptake values (SUVmax) of 9.2 and 5.1, respectively. Both patients underwent surgical resection, with pathology confirming mediastinal EHEs.

**Conclusion:**

Mediastinal EHE presents as a well-defined soft-tissue mass with punctate calcifications and heterogeneous enhancement, typically located in the anterior mediastinum with invasion into medium or large veins. Moreover, it should be considered in the differential diagnosis of mediastinal tumors.

## 1 INTRODUCTION

Epithelioid hemangioendothelioma (EHE), first described by Weiss and Enzinger in 1982 [1, 2], is a rare vascular neoplasm exhibiting clinical and pathological characteristics intermediate between benign hemangiomas and malignant angiosarcomas.

EHE often occurs in the lung, bone, and liver, accounting for over 65% of cases, whereas mediastinum involvement is extremely rare [3, 4]. Despite a few reported cases, radiological features of mediastinal EHE remain poorly understood. Here, we present two cases of mediastinal EHE with detailed CT, MRI, and PET-CT imaging features.

## 2 CASE PRESENTATION

Two middle-aged female patients, Case 1 (39 years old) and Case 2 (49 years old), presented with a 2-month history of cough. Additionally, Case 2 reported chest tightness and shortness of breath. Contrast-enhanced CT revealed well-defined low-density masses in the right anterior superior mediastinum, measuring 3.9 cm × 2.8 cm and 3.1 cm × 2.2 cm, respectively, with distinct characteristics. Case 1 presented an oval-shaped mass with mild enhancement, invading the brachiocephalic vein-superior vena cava junction, associated with azygos vein dilation and right diaphragm elevation. In contrast, Case 2 exhibited a markedly heterogeneous mass with intralesional spotty calcifications and necrotic areas, demonstrating significant enhancement. Both cases shared similar MRI features, showing isointense on T1WI, slightly hyperintense on T2WI, and partial diffusion restriction on DWI, while demonstrating divergent enhancement characteristics: moderate heterogeneous enhancement in Case 1 and significant enhancement in Case 2. PET-CT detected significant FDG uptake in both masses, with differing SUVmax values (9.2 vs 5.1). The imaging features of the two cases are shown in Figs. (**1** and **2**). Both patients underwent surgical resection of the lesions, and postoperative pathology confirmed the diagnosis of mediastinal EHE. This study was approved by the Ethics Committee of Zhongshan Hospital Fudan University, and the approval number is [B2025-100].

## 3 DISCUSSION

Epithelioid hemangioendotheliomas (EHEs) are rare vascular endothelial neoplasms that predominantly occur in soft tissues, bone, lungs, and liver, with mediastinal involvement being exceptionally rare [1]. According to the literature, most mediastinum EHEs are located in the anterior or upper mediastinum, appearing as well-defined, oval, or spherical-shaped masses, typically exhibiting uniformly low density. Approximately 50% of the cases demonstrate intratumoral calcifications, which histopathologically correlate with metaplastic bone formation, osteoclast-like giant cells, or phleboliths [5]. This correlation was confirmed in our second case, where punctate calcifications corresponded histologically to osteoclast-like giant cells. The co-existence of calcific and adipose components within mediastinal lesions may precipitate diagnostic confusion with teratoma [5, 6]. Critical imaging differentiation lies in calcification morphology: EHE typically presents with scattered punctate calcification, whereas teratoma characteristically displays coarse calcifications with tooth-like or osseous configurations [7]. Adipose components in mediastinal EHE are believed to result from the encasement of surrounding tissues during tumor growth, though they were not observed in our cases. As vascular-centric neoplasms, more than 50% of mediastinal EHEs demonstrate direct vascular engagement, predominantly involving major venous structures, such as the superior vena cava or brachiocephalic veins, an important imaging feature that aids differential diagnosis [8].

MRI features of mediastinal EHE have rarely been reported, but most cases show isointensity on T1WI and hypo- to iso-hyperintensity on T2WI [9]. The presence of partially restricted diffusion on DWI, reflecting high cellular density and malignant potential, serves as a valuable discriminator from benign mediastinal tumors like teratomas and thymomas. While heterogeneous enhancement is a characteristic feature of mediastinal EHE, its intensity demonstrates considerable variability, ranging from mild to significant enhancement. This variability was evident in our cases, with Case 1 showing mild enhancement contrasting with significant enhancement observed in Case 2. Consequently, enhancement patterns alone lack sufficient specificity to serve as a definitive diagnostic criterion for mediastinal EHE.

Most mediastinal EHEs demonstrate FDG activity on PET, with reported SUVmax values ranging from 6.5 to 14.7 [10-12]. This metabolic profile indicates the tumor’s intermediate-grade biological behavior, positioning it between benign lesions and aggressive malignancies. In addition, PET/CT demonstrates superior sensitivity to CT or MRI for both tumor staging and detection of multicentric EHEs or metastatic involvement [13, 14].

The differential diagnosis of mediastinal EHE primarily includes thymoma, thymic carcinoma, thyroid tumor, lymphoma, and germ cell tumors [15]. Accurate preoperative diagnosis remains challenging, as mediastinal EHE is often misdiagnosed. A well-defined soft-tissue mass in the anterior mediastinum with punctate calcifications, heterogeneous enhancement, and medium to large vein invasion should raise suspicion for EHE. Multimodal imaging helps reveal its characteristics from various perspectives and aids in accurate diagnosis.

## CONCLUSION

In conclusion, we presented two rare cases of mediastinal EHE, a tumor that is challenging to distinguish from other mediastinal tumors. Characteristic imaging manifestations include a well-demarcated anterior mediastinal soft-tissue mass demonstrating three key features: (1) punctate calcifications, (2) heterogeneous enhancement patterns, and (3) vascular engagement with medium-to-large venous structures. Furthermore, multimodal imaging is valuable for differential diagnosis.

## Figures and Tables

**Fig. (1):**
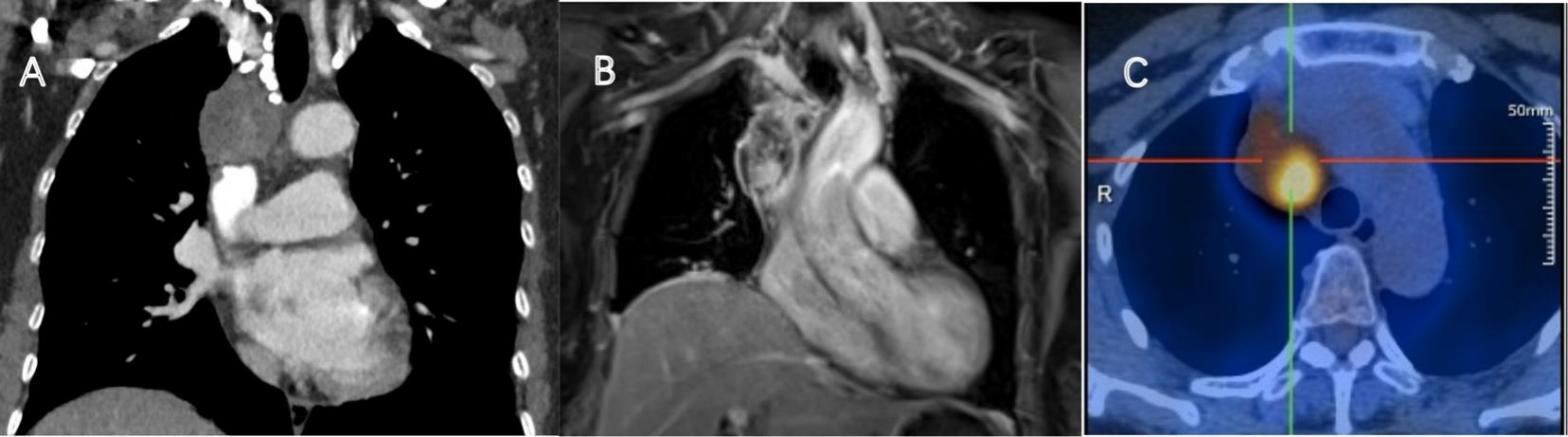
Multimodal images of a 39-year-old woman with a right anterior superior mediastinal mass. Coronal-enhanced CT revealed tumor invasion at the brachiocephalic vein-superior vena cava junction (**A**), MRI showed moderately heterogeneous contrast enhancement (**B**), and PET-CT detected hypermetabolic activity in the upper mediastinum (SUVmax = 9.2) (**C**).

**Fig. (2):**
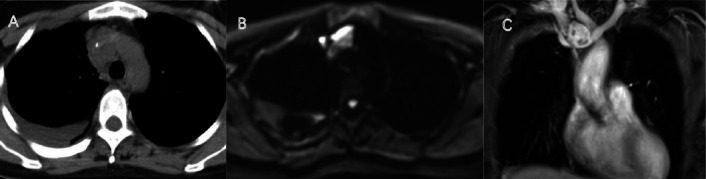
A 49-year-old woman showed a well-defined low-density mass in the right anterior superior mediastinum on axial CT with spotty calcifications (**A**); DWI sequence showed partial diffusion restriction (**B**), and enhanced MRI revealed heterogeneous enhancement with non-enhancing necrotic regions (**C**).

## Data Availability

All data generated or analyzed during this study are included in this published article.

## LIST OF ABBREVIATIONS

EHE: Epithelioid Hemangioendothelioma
SVC: Superior Vena Cava

## STANDARDS OF REPORTING

CARE guidelines were followed.
